# Supplementary material for: Rapid transmission and tight bottlenecks constrain the evolution of highly transmissible SARS-CoV-2 variants
Source: Nat Commun. 2023 Jan 17;14:272. doi: 10.1038/s41467-023-36001-5 (PMC9844183; doi:10.1038/s41467-023-36001-5)
Supplement: Supplementary file 1 — Supplementary Information [file 41467_2023_36001_MOESM1_ESM.pdf]

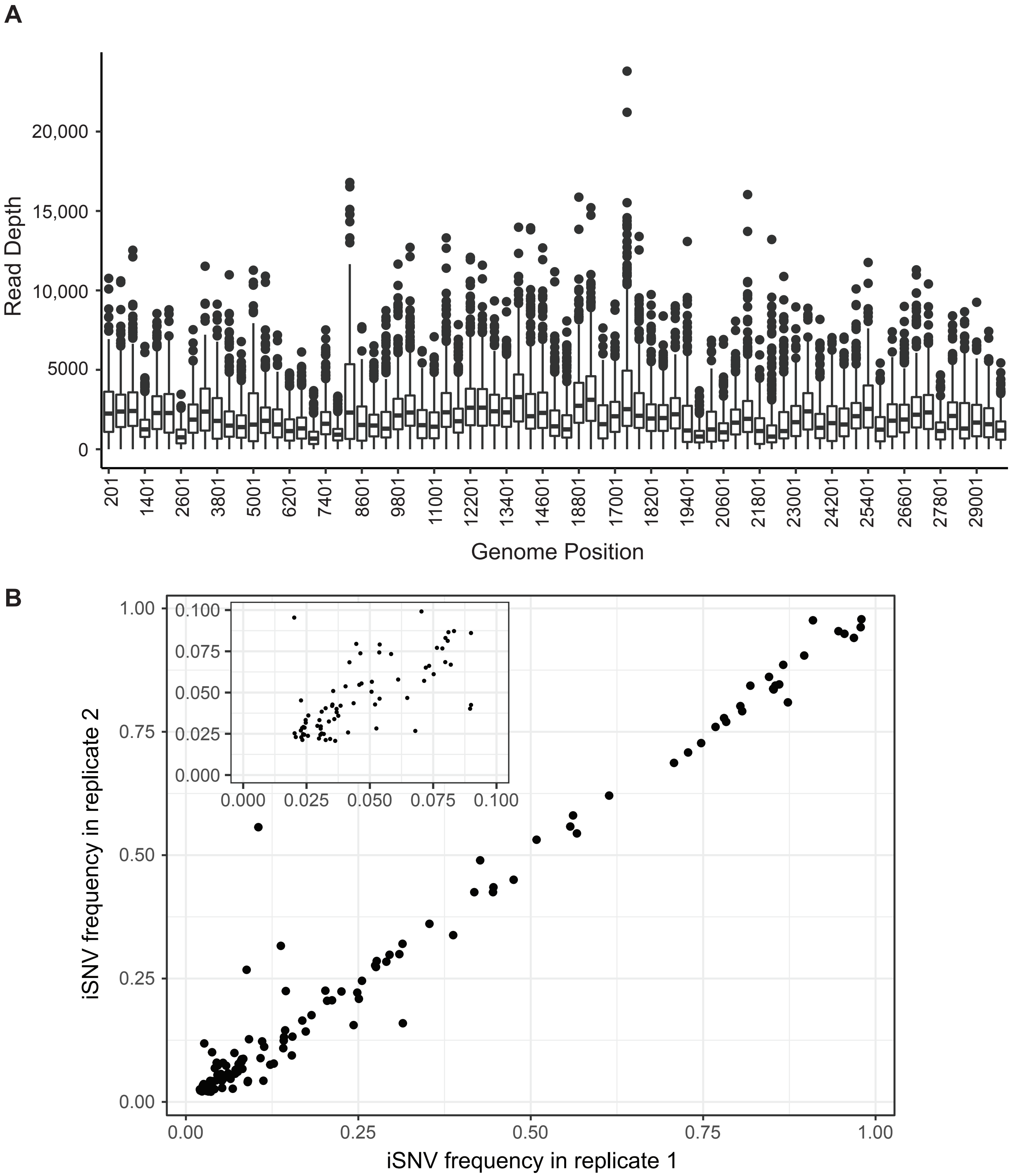

Supplementary Figure 1. Sequencing coverage and consistency. (A) Boxplot of median (+/- IQR) coverage across the genome in 400bp non-overlapping sliding windows. (B) Frequency of iSNV in each replicate for iSNV that were identified in both replicates. The inset shows replicate frequencies between 0 and 0.1.

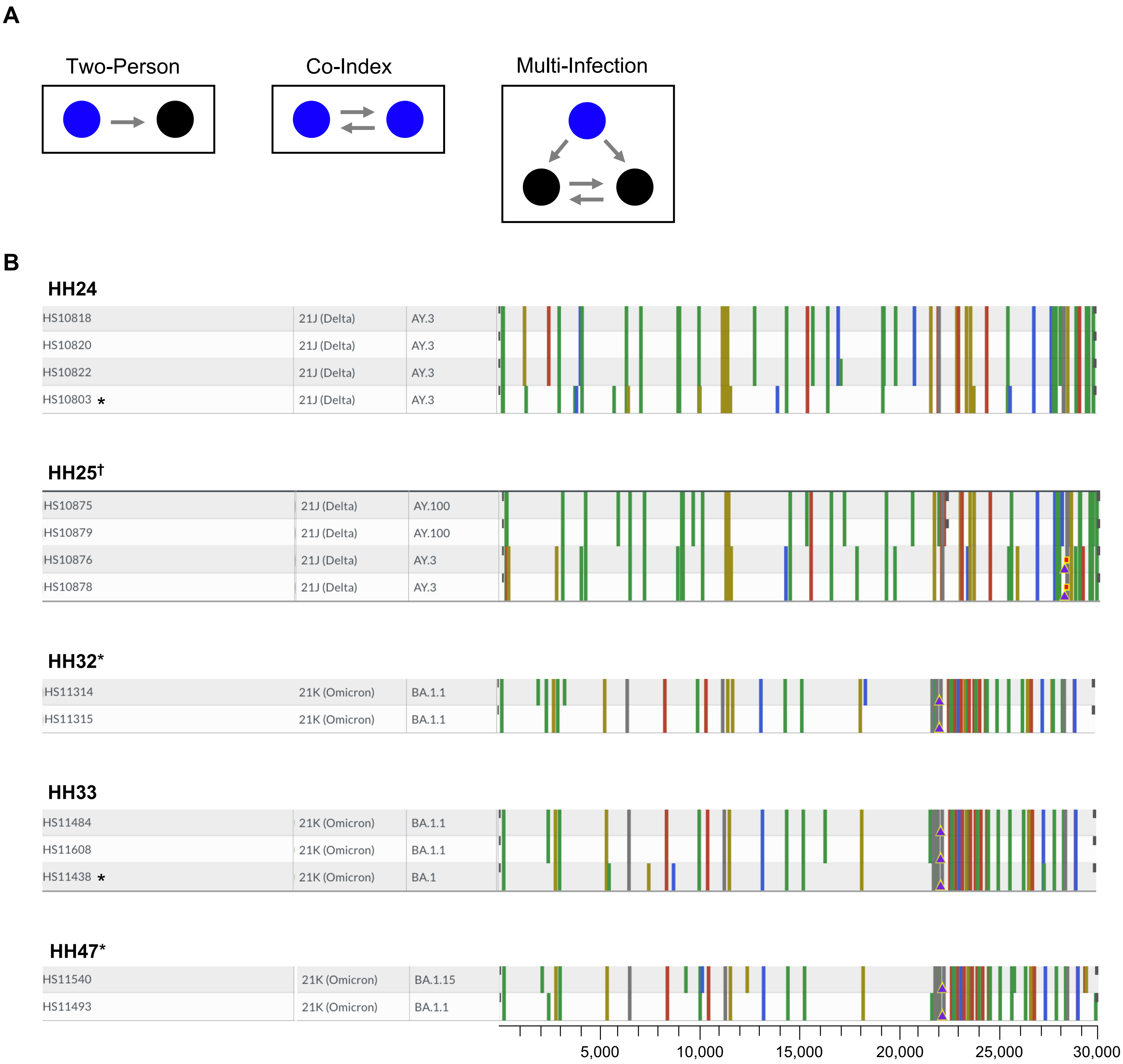

Supplementary Figure 2. Inclusion and exclusion of transmission pairs. (A) Examples of possible transmission pairs in households. In each panel, the index cases are in blue, and the household contacts are in black. The grey arrows indicate transmission pairs, and they point from the donor to the recipient. (B) Consensus genome alignments inconsistent with household transmission. The genomes were visualized using Nextclade. Both Nextclade and Pango lineages are reported. Colored bars are mutations with reference to the Wuhan-Hu-1/2019 (MN908947) strain. Approximate genome positions are indicated below figure. Gray partial bars indicate missing data. Asterisks next to household names indicate households that were removed from further analyses. Asterisks next to sample names indicate samples that were removed from further analyses, while the rest of the household was retained. The black cross (†) indicates a household with two separate transmission pairs.

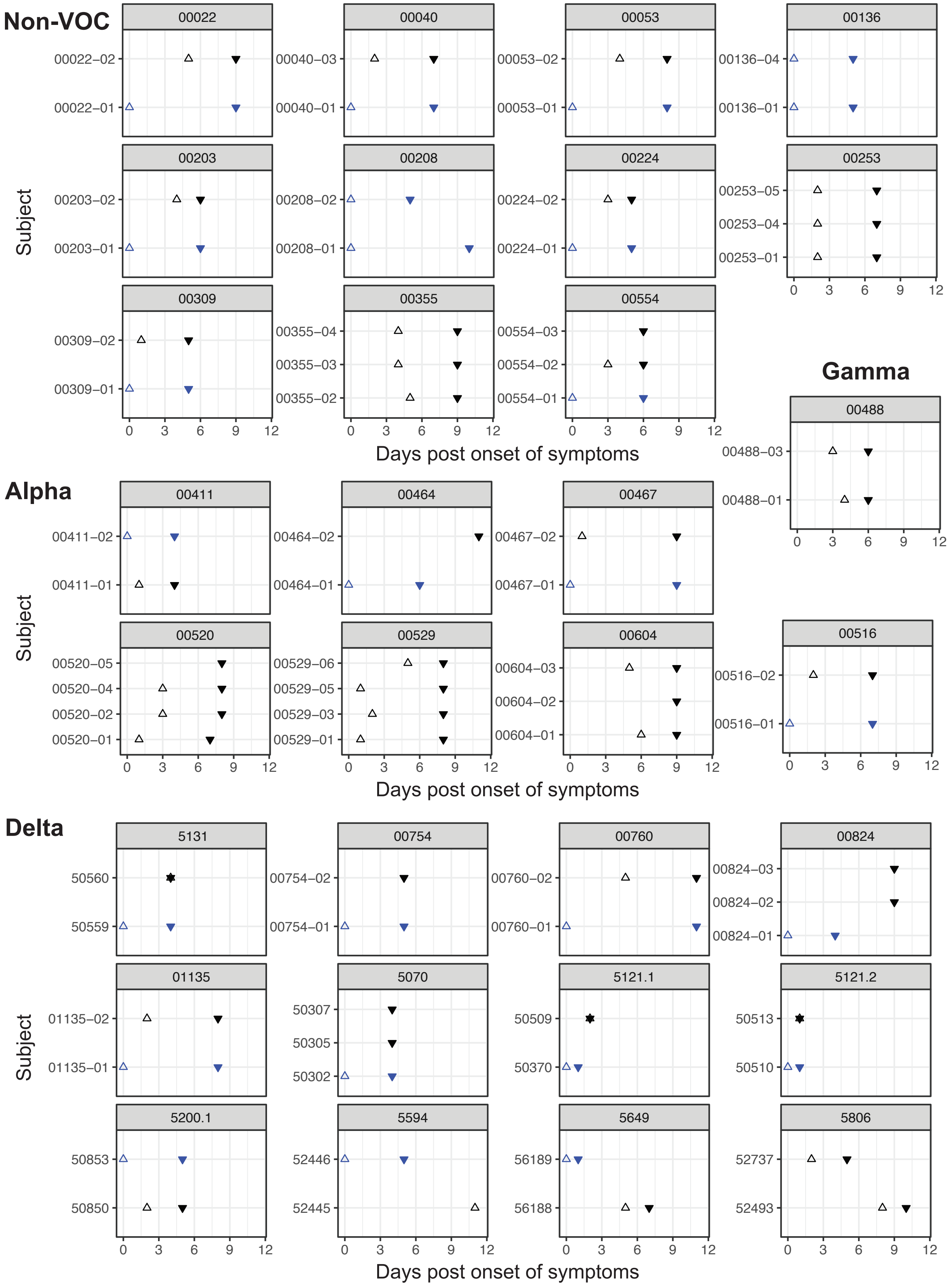

Supplementary Figure 3. Timing of symptom onset and specimen collection by household. Each panel shows a household, grouped by the indicated clades. Within each household, blue symbols indicate index case(s) and black symbols indicate household contact(s). Open triangles indicate time of symptom onset and filled triangles indicate specimens that were sequenced. When symptom onset and sample collection occur on the same day, overlapping triangles form a star. If there is no symptom onset, the case was considered to be asymptomatic.

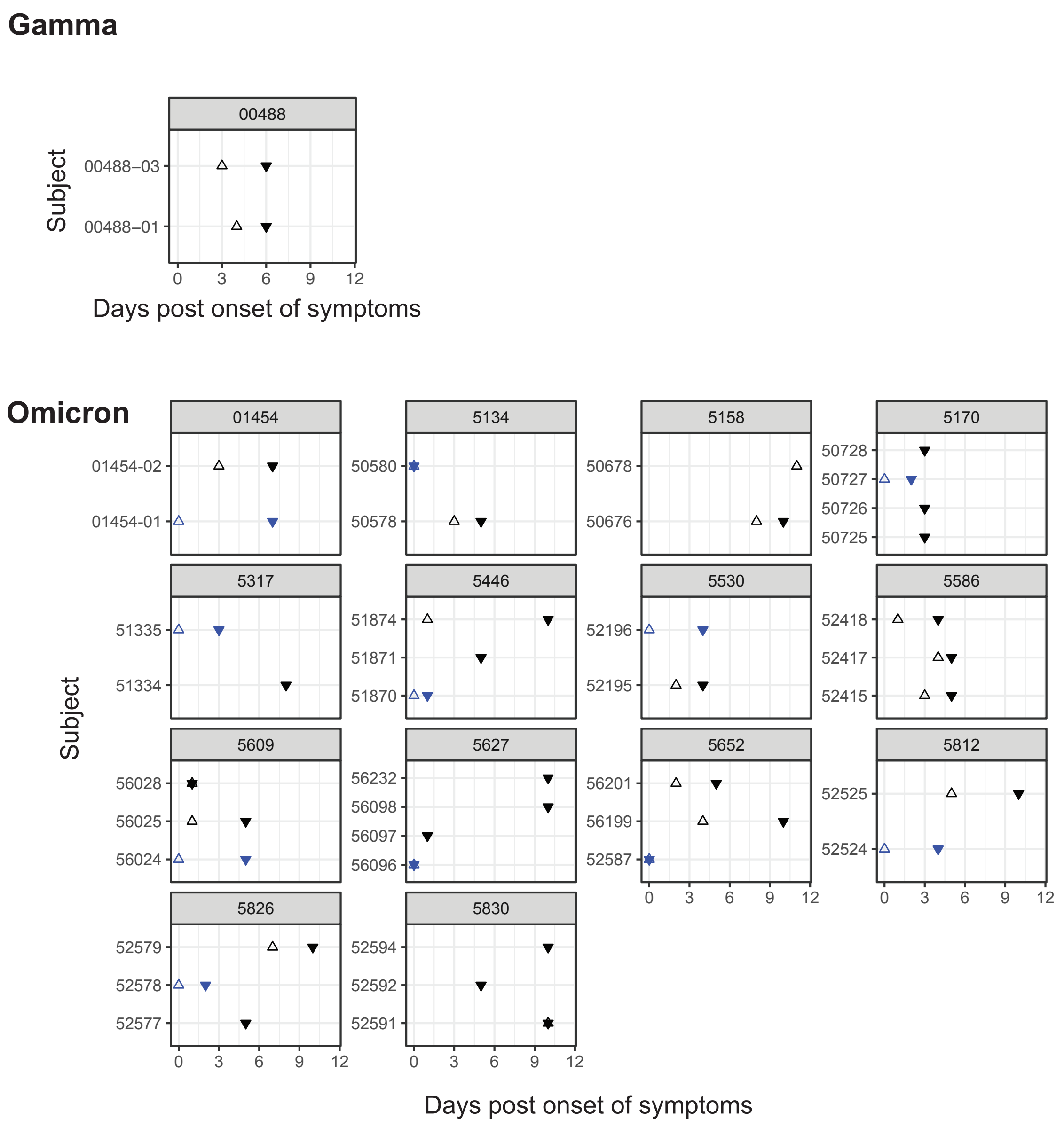

Supplementary Figure 4. Timing of symptom onset and specimen collection by household. Each panel shows a household, grouped by the indicated clades. Within each household, blue symbols indicate index case(s) and black symbols indicate household contact(s). Open triangles indicate time of symptom onset and filled triangles indicate specimens that were sequenced. When symptom onset and sample collection occur on the same day, overlapping triangles form a star. If there is no symptom onset, the case was considered to be asymptomatic.

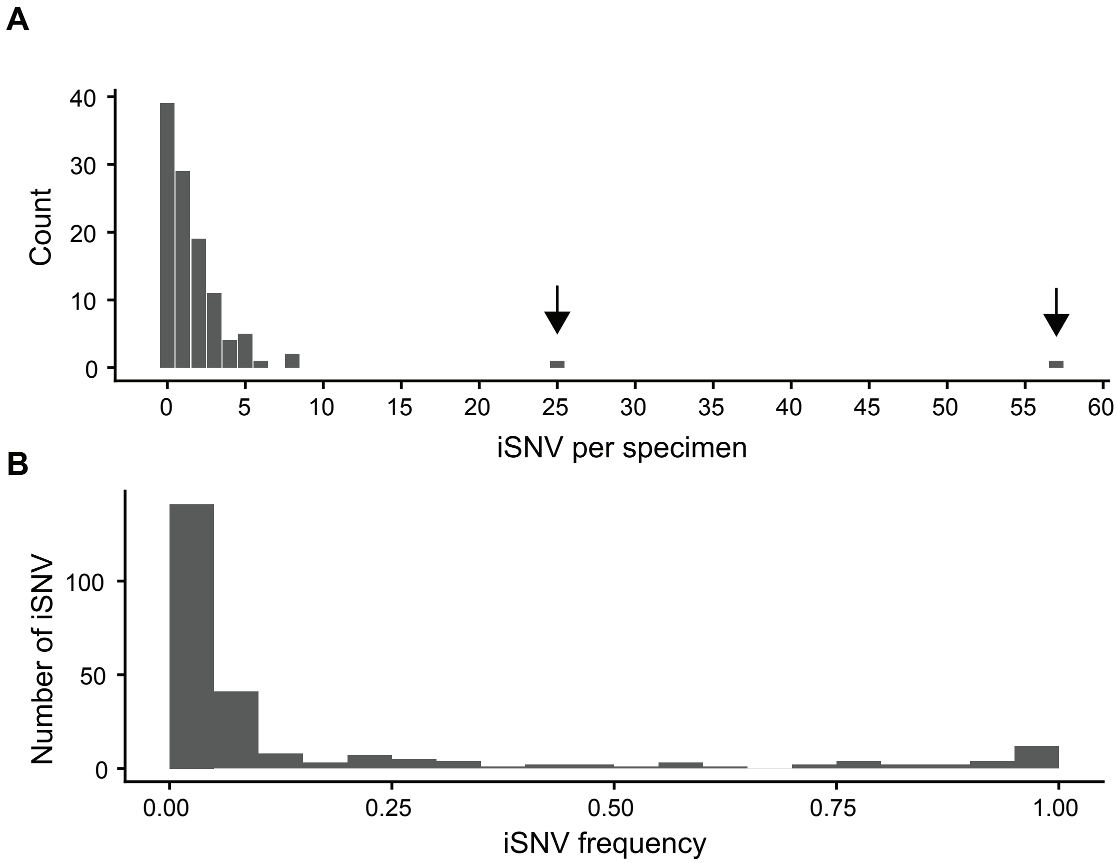

Supplementary Figure 5. iSNV detected when sequencing replicates are merged. (A) The number of iSNV per specimen. Nearly all still had a low number of iSNV. However, merging the reads greatly increased the number of iSNV in two individuals. These iSNV were near the 2% threshold. (B) Most iSNV were found at low frequencies. The frequency distribution shifted toward lower frequencies compared to when iSNV had to be detected in both replicates.
