# Supplementary material for: Rapid transmission and tight bottlenecks constrain the evolution of highly transmissible SARS-CoV-2 variants
Source: Nat Commun. 2023 Jan 17;14:272. doi: 10.1038/s41467-023-36001-5 (PMC9844183; doi:10.1038/s41467-023-36001-5)
Supplement: Supplementary file 3 — Description of Additional Supplementary Files [file 41467_2023_36001_MOESM3_ESM.docx]

Description of Additional Supplementary Files

File Name: Supplementary_Data.xlsx

Description: File contains four tabs for Supplementary Data 1-4

Supplementary Data 1: Household sizes and numbers of possible transmission pairs

Supplementary Data 2: Bottleneck sizes by household and transmission pair for technical duplicate dataset

Supplementary Data 3: Bottleneck sizes by household and transmission pair for merged read dataset

Supplementary Data 4: Genomic sites masked from analysis as described in Methods
